# Disseminated Melioidosis Complicated by Prostatic Abscess and Splenic Involvement: Diagnostic and Therapeutic Insights

**DOI:** 10.7759/cureus.69961

**Published:** 2024-09-22

**Authors:** Krishna Geetha Narne, Jagadeswar Kakumani, Vaishnavi K. I. S. N., Vivekanandan T, Gowrishankar A

**Affiliations:** 1 Internal Medicine, Saveetha Medical College and Hospital, Saveetha Institute of Medical and Technical Sciences, Saveetha University, Chennai, IND; 2 General Medicine, Saveetha Medical College and Hospital, Saveetha Institute of Medical and Technical Sciences, Saveetha University, Chennai, IND

**Keywords:** burkholderia pseudomallei, diabetes mellitus, immunocompromised, melioidosis, prostatic abscess, spleenic abscess, splenomegaly

## Abstract

This case report details the clinical course of a 53-year-old male farmer with a 15-year history of diabetes mellitus who presented with a 20-day history of pyrexia, rigors, and shivering, as well as problems in the urogenital system and left hypochondrial pain. Notably, he had been diagnosed with spinal tuberculosis, which was successfully treated five years ago. On evaluation, there was tenderness in the suprapubic region as well as the left hypochondrium; moreover, rectal examination showed that the prostate was boggy and tender. The laboratory tests revealed microcytic hypochromic anemia, increased inflammatory markers, and uncontrolled diabetes. Imaging studies reported splenomegaly containing multiple low-density lesions accompanied by cystitis and a prostatic abscess. Positive blood culture samples indicated *Burkholderia pseudomallei,* thereby signifying disseminated melioidosis. He underwent cystoscopy followed by prostatic deroofing and received intravenous meropenem and prolonged oral cotrimoxazole treatment thereafter. Within 10 days after the initiation of treatment, significant symptomatic relief was achieved. This report highlights the need for a high index of suspicion for melioidosis in systemic infection patients with diabetes and emphasizes prompt surgical intervention along with appropriate medical therapy in complex cases, especially those involving a non-clear-cut diagnosis or severe disease presentation.

## Introduction

Melioidosis is a bacterial infection that *Burkholderia pseudomallei* causes, which can be fatal in many cases. The disease is usually referred to as a “great mimicker” due to a myriad of clinical presentations that vary from acute septicemia to chronic abscess formation, thereby making its diagnosis difficult [1]. The microbes are commonly obtained through direct contact with contaminated soil or water, and individuals having pre-existing diseases such as diabetes mellitus, immunosuppression, or chronic renal disease are more vulnerable to infection [2]. Diabetes mellitus is the most remarkable predisposing factor for melioidosis, leading to an approximately 12-fold increase in infection risk [3]. Diabetics are more prone because of their impaired immune responses, resulting in severe forms such as dissemination of this illness [4]. We present herein a complex case of disseminated melioidosis complicated by treated spinal tuberculosis in a 53-year-old man who had a history of type 2 diabetes mellitus. His symptoms included systemic symptoms like prolonged fever, urogenital complaints, and abdominal pains, which were later connected with splenic abscesses and prostatic abscesses. This case highlights the need for considering melioidosis, especially in those patients living in endemic areas known to have predisposing factors such as diabetics. Prompt identification and initiation of appropriate antibiotic therapy play a crucial role in managing this disease since it may lead to fatalities if left untreated [5]. Similarly, this report emphasizes imaging modalities and microbiological studies for confirmation purposes, thus underscoring difficulties encountered during the diverse clinical courses of melioidosis. Given the patient’s history of spinal tuberculosis, there was a wide differential diagnosis necessitating a careful, comprehensive approach towards both diagnosis and management. This successful outcome illustrates how multidisciplinary approaches should be used when treating complicated infections among immunocompromised hosts.

## Case presentation

A 53-year-old male patient, a farmer by occupation from South Asia, has been suffering from diabetes for 15 years and is compliant with consistent treatment. The patient presented to the clinic with a high-grade fever associated with chills and rigors persisting for 20 days. He also complained of pain in the groin, discomfort during micturition, constipation, and dull, aching abdominal pain in the left hypochondrium, which had started 15 days before he came to the hospital. Besides this, he had dizziness, fatigue, and easy tiring. His medical history includes spinal tuberculosis diagnosed five years ago, which was treated with antitubercular therapy. On physical examination, there was tenderness over the left hypochondrium with mild suprapubic pain and mild splenomegaly. The prostate felt like a boggy prostate on digital rectal examination grade two and was tender, suggestive of significant prostatic involvement indicating inflammation or infection due to conditions such as acute prostatitis or prostatic abscess. Other systems were within normal limits. Blood investigations (Table 1) showed microcytic hypochromic anemia, low normal platelet count and WBC counts, and significant elevation in ESR at 120 mm/hr and CRP at 60 mg/L but later improved after treatment. His liver function tests were within normal range except for gamma-glutamyl transferase (287 U/L) and alkaline phosphatase (448 U/L). Renal function tests and electrolytes were normal. Serum iron was severely reduced (<10 μg/dl), while HbA1c was elevated at 9%, indicating uncontrolled type 2 diabetes mellitus. Serum prostate-specific antigen level was <1 ng/ml.

**Table 1:** Laboratory values Hb: hemoglobin, RBC: red blood cell, PCV: packed cell volume, MCV: mean corpuscular volume, MCH: mean corpuscular hemoglobin, MCHC: mean corpuscular hemoglobin concentration, TLC: total leukocyte count, ANC: absolute neutrophil count, ESR: erythrocyte sedimentation rate, CRP: C-reactive protein, AST: aspartate transaminase, ALT: alanine transaminase, ALKP: alkaline phosphatase, GGT: gamma-glutamyl transferase, HBA1C: glycosylated Hb, FBS: fasting blood sugar, PPBS: postprandial blood sugar, PSA: prostate-specific antigen, CBNAAT: cartridge-based nucleic acid amplification test, TIBC: total iron binding capacity, HPE: histopathological examination, C/S: culture and sensitivity, KOH: potassium hydroxide, TB: tuberculosis, MGIT: mycobacteria growth indicator tube

Investigation	Patient’s value	Reference value
Hb (g/dL)	8.9	Male 13-17
Total RBC count (million/cu.mm)	3.93	Male 4.5-5.5
PCV (%)	28.4	Male 40-50
MCV (fl)	72.3	83-101
MCH (pg)	22.6	27-32
MCHC (g/dl)	31.3	31.5-34.5
Red cell distribution width (%)	18.1	11.6-14
Platelet count (lakhs/cu mm)	1.80	1.5-4.5
TLC (cells/cumm)	5970	4000-10000
Neutrophils (%)	79.2	40-80
Lymphocytes (%)	16.9	20-40
Monocytes (%)	3.7	2-10
Eosinophils (%)	0.0	1-6
Basophils (%)	0.2	<1-2
ANC (cells/cu.mm)	4730	2000-7000
ESR (mm/hr)	120	Male 17-60 years: <12
CRP mg/L	60.3	<10 mg/L - negative (OR), >10 mg/L - positive
Serum sodium (meq/L)	130	137-145
Serum potassium (meq/L)	4.3	3.5-5
Serum chloride (meq/L)	90	96-106
Serum bicarbonate (meq/L)	16.1	22-26
Urea (mg/dl)	31	17-43
Creatinine (mg/dl)	0.6	0.7-1.4
Uric acid (mg/dl)	3.2	2.5-7
Total bilirubin (mg/dl)	1.25	0.2-1.3
Direct bilirubin (mg/dl)	0.93	0.1-0.4
Total protein (g/dl)	8.5	6-8
AST (IU/L)	33	14-36
ALT (IU/L)	52	5-50
ALKP (IU/L)	472	3.5-5
Albumin (g/dl)	3.7	3.5-5
GGT	268	<55
HBA1C	9	<5.7%
FBS (mg/dl)	225	70-100
PPBS (mg/dl)	353	80-140
Urine routine	Glucose: +++, leukocyte: present	Absent
Total PSA	1.016	<4 ng/ml
Urine culture	No bacteriuria	-
Blood culture	*Burkholderia pseudomallei* (susceptible to imipenem, meropenem, cotrimoxazole)	-
Peripheral smear	*Normocytic normochromic*/microcytic hypochromic anemia	-
GeneXpert - CBNAAT	MTB - not detected	-
Serum Iron (mcg/dl)	<10	37-170
TIBC (mcg/dl)	308	Male 261-462
Ferritin (mcg/dl)	265	Male 17.9-464
Histopathological examination (HPE) of prostatic abscess	Focal granulomatous inflammation	-
Fungal C/S	No fungal growth in culture	-
KOH mount	Negative for fungal elements	-
TB culture (MGIT)	No growth	-

**Figure 1:**
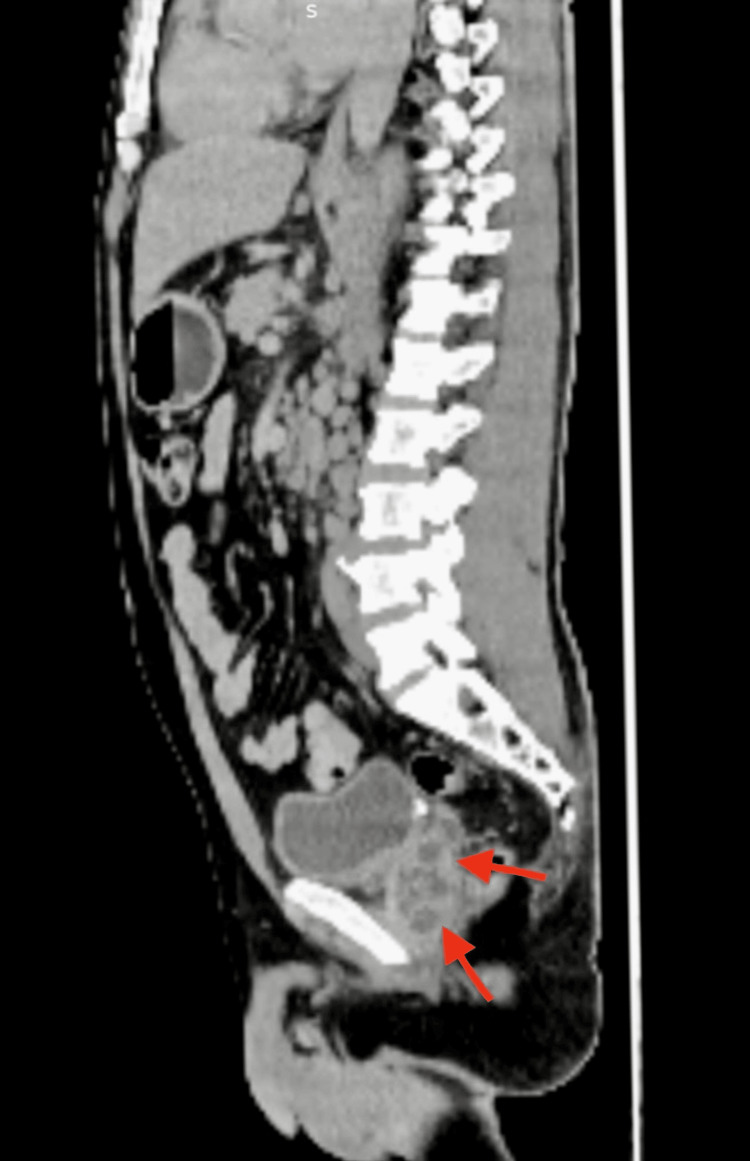
Sagittal section of CECT abdomen showing multiple hypodense lesions in the prostate (arrows) CECT: contrast-enhanced computed tomography

**Figure 2:**
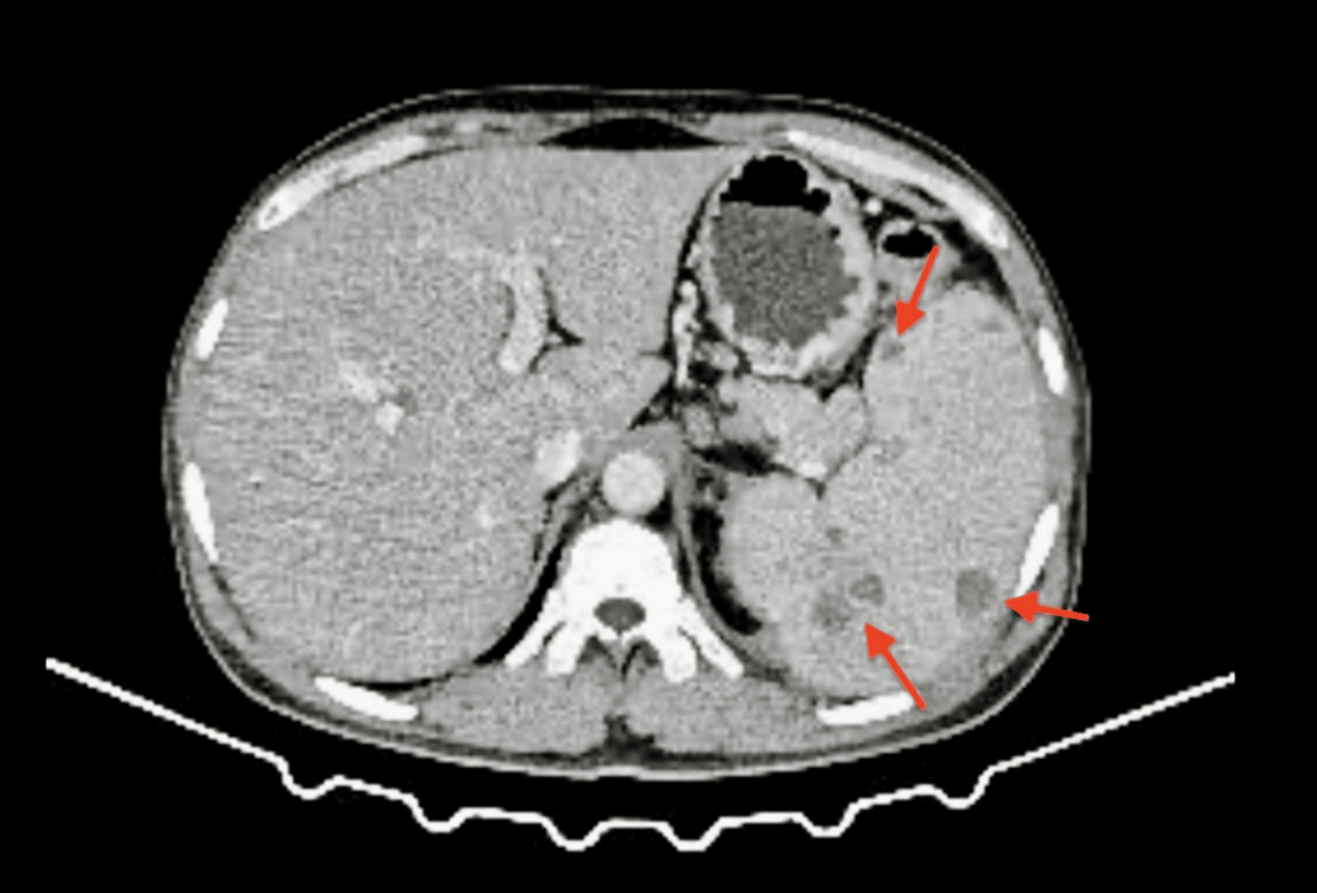
Axial section of the CECT abdomen showing multiple hypodense lesions in the spleen (arrows) CECT: contrast-enhanced computed tomography

On electrocardiography, multiple ventricular premature complexes (VPCs) were seen with poor R wave progression (PRWP) in leads V1-V4, whereas abdominal USG revealed splenomegaly along with few hypoechoic lesions in the spleen and cystitis. A CT scan of the kidneys, ureters, and bladder showed mild perinephric and periureteric fat stranding, cystitis, and prostatic enlargement with heterogeneous attenuation, which may indicate a prostatic abscess. Further imaging with CECT abdomen confirmed a prostatic abscess (Figure 1) and splenomegaly with multiple fairly defined hypodense, peripherally enhancing foci of varying sizes in the spleen (Figure 2) along with peritoneal and retroperitoneal lymphadenopathy suggestive of infective pathology.

**Figure 3:**
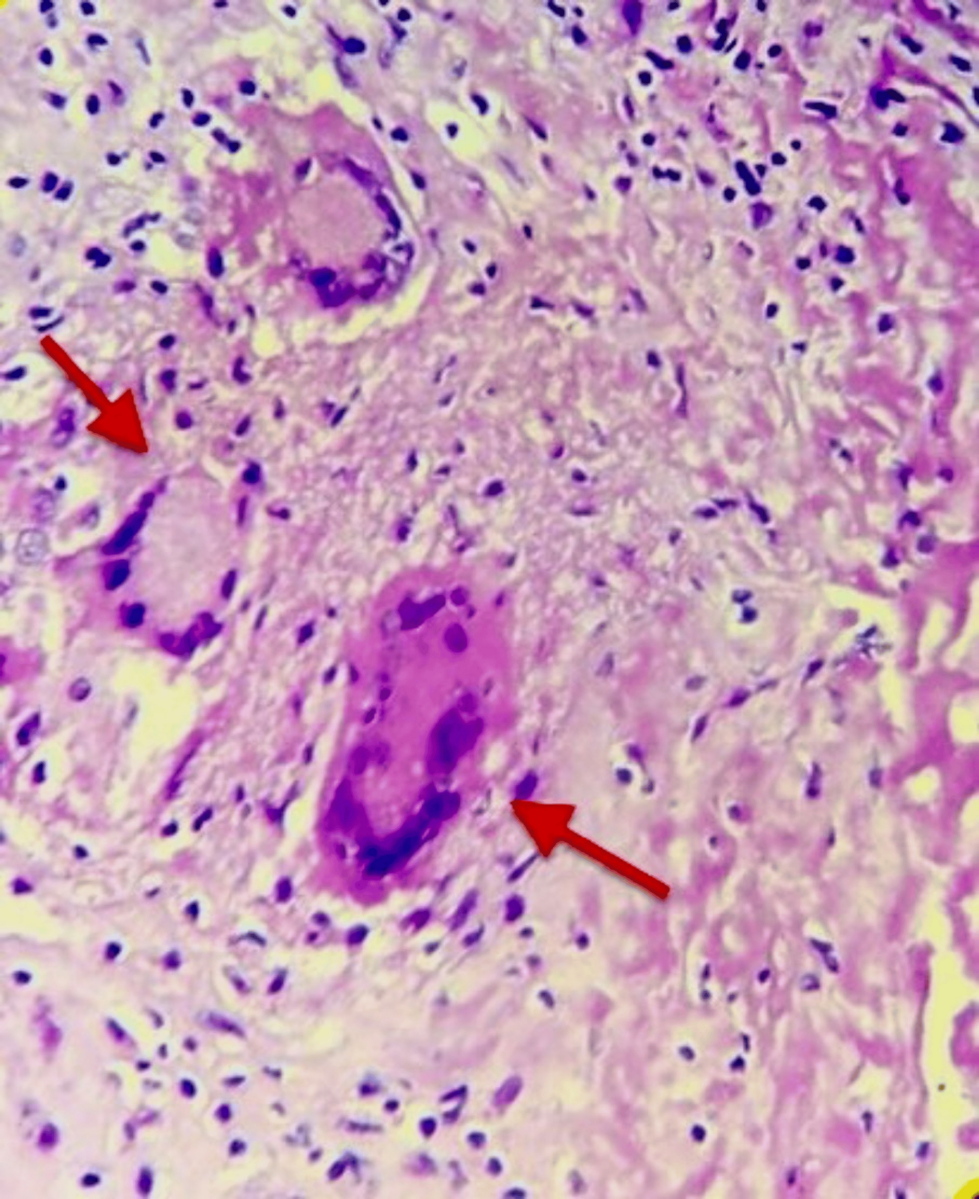
HPE showing granuloma (arrows) HPE: epithelioid hemangioendothelioma

The causative organism found in the blood culture was *Burkholderia pseudomallei*. There was no growth on fungal/urine cultures. Histopathological examination (HPE; Figure 3) of a specimen from the prostatic abscess revealed focal granulomatous inflammation, whereas staining for acid-fast bacilli (AFB), cartridge-based nucleic acid amplification test (CBNAAT), and mycobacteria growth indicator tube (MGIT) culture were negative for tuberculosis.

A surgical intervention comprising a deroofing procedure of the prostate gland through cystoscopy was performed via transurethral resection of the prostate using the Maureyer resecting technique. A total of 20-30 g of the prostate tissue was resected, followed by catheter irrigation. Initially, the patient received intravenous meropenem 1 g three times a day for 14 days, followed by oral cotrimoxazole (trimethoprim-sulfamethoxazole) at a double strength of 160/800 mg for six months. He showed symptomatic improvement over 10 days after starting treatment.

## Discussion

This is a challenging diagnostic and therapeutic situation involving a patient with diabetes mellitus who has a complicated urogenital infection, systemic involvement, and a significant history of tuberculosis. The diagnosis of melioidosis caused by *Burkholderia pseudomallei* is supported by the clinical picture, radiological findings, and blood culture results. *Burkholderia pseudomallei* has an incidence rate of 5.0 per 100,000 people at risk per year. Melioidosis is a contagious illness that regularly affects individuals with predisposing factors such as diabetes mellitus in this patient [6]. Multiple hypo-dense lesions within the spleen, along with positive blood cultures for *Burkholderia pseudomallei* are indicative of disseminated melioidosis. This organism causes various clinical conditions ranging from localized abscesses to serious systemic infections, particularly in those who have impaired immunity [7]. The high ESR and CRP levels reflect persistent inflammation, while anemia and low serum iron levels could be due to chronic disease and underlying infection [8]. The presence of multiple VPCs and PRWP on ECG may suggest underlying ischemic changes or stress cardiomyopathy, likely worsened by systemic infection [9]. Prostatic abscesses are an uncommon manifestation but can occur in association with diabetes mellitus and chronic infection [10]. It was, however, important to get concerned about tubercular reactivation because we found granulomatous focal inflammation on HPE of prostatic abscess even though AFB, CBNAAT, and MGIT culture tests were negative, hence making it less probable. Other differential diagnoses like malignancies like lymphoma, fungal infections, nocardiosis, HIV, Q fever, and brucellosis are to be ruled out. Surgical management, which included cystoscopy as well as prostatic deroofing, was hence indispensable in draining the abscess while decreasing the burden of sepsis. Meropenem followed by long-term cotrimoxazole therapy was, therefore, selected in line with standard treatment protocols for melioidosis to ensure complete eradication and prevent relapse.

## Conclusions

This case highlights the importance of including melioidosis as a potential diagnosis in diabetic patients with a widespread infection, particularly in regions where the disease is common. Early-stage diagnosis and intensive therapy are crucial for a favorable outcome in individuals with multi-organ illness. The presence of both spinal tuberculosis and uncontrolled diabetes in the patient's medical history adds complexity to the clinical situation, necessitating a multidisciplinary approach to care. Furthermore, this specific instance exemplifies the efficacy of customized antimicrobial treatment in conjunction with suitable surgical intervention.
